# The cat as a naturally occurring model of renal interstitial fibrosis: Characterisation of primary feline proximal tubular epithelial cells and comparative pro-fibrotic effects of TGF-β1

**DOI:** 10.1371/journal.pone.0202577

**Published:** 2018-08-23

**Authors:** Jack S. Lawson, Hui-Hsuan Liu, Harriet M. Syme, Robert Purcell, Caroline P. D. Wheeler-Jones, Jonathan Elliott

**Affiliations:** 1 Comparative Biomedical Sciences, The Royal Veterinary College, London, United Kingdom; 2 Clinical Sciences and Services, The Royal Veterinary College, North Mymms, Hatfield, Herts, United Kingdom; University Medical Center Utrecht, NETHERLANDS

## Abstract

Chronic kidney disease (CKD) is common in both geriatric cats and aging humans, and is pathologically characterised by chronic tubulointerstitial inflammation and fibrosis in both species. Cats with CKD may represent a spontaneously occurring, non-rodent animal model of human disease, however little is known of feline renal cell biology. In other species, TGF-β1 signalling in the proximal tubular epithelium is thought to play a key role in the initiation and progression of renal fibrosis. In this study, we first aimed to isolate and characterise feline proximal tubular epithelial cells (FPTEC), comparing them to human primary renal epithelial cells (HREC) and the human proximal tubular cell line HK-2. Secondly, we aimed to examine and compare the effect of human recombinant TGF-β1 on cell proliferation, pro-apoptotic signalling and genes associated with epithelial-to-mesenchymal transition (EMT) in feline and human renal epithelial cells. FPTEC were successfully isolated from cadaverous feline renal tissue, and demonstrated a marker protein expression profile identical to that of HREC and HK-2. Exposure to TGF-β1 (0–10 ng/ml) induced a concentration-dependent loss of epithelial morphology and alterations in gene expression consistent with the occurrence of partial EMT in all cell types. This was associated with transcription of downstream pro-fibrotic mediators, growth arrest in FPTEC and HREC (but not HK-2), and increased apoptotic signalling at high concentrations of TGF- β1. These effects were inhibited by the ALK5 (TGF-β1RI) antagonist SB431542 (5 μM), suggesting they are mediated via the ALK5/TGF-β1RII receptor complex. Taken together, these results suggest that TGF-β1 may be involved in epithelial cell dedifferentiation, growth arrest and apoptosis in feline CKD as in human disease, and that cats may be a useful, naturally occurring model of human CKD.

## Introduction

Chronic kidney disease (CKD) is common in geriatric cats, with a reported prevalence of 28–50% [1, 2]. The majority of cats with CKD have non-specific renal lesions and the predominant morphological diagnosis in these cases is chronic tubulointerstitial inflammation and fibrosis [3, 4]. CKD is similarly common in humans [5, 6], with end-stage kidney disease also characterised by tubulointerstitial fibrosis, despite differing aetiology [7]. Whilst fibrosis is a normal sequelae of injury, it is thought that in CKD the normal wound healing response fails to terminate [8, 9] and the expansion of the extra-cellular matrix (ECM) gradually destroys normal tissue structure [10].

In cats with naturally occurring CKD, hyperphosphataemia [3] and proteinuria [3, 11] correlate with severity of renal fibrosis, and these factors are also known to be risk factors for the progression of renal disease and mortality [12–14]. Recently, a study inducing renal ischaemia in cats has also provided evidence to suggest that renal hypoxia may play a role in the development of renal fibrosis [15]. A cell culture model of the feline tubular epithelium would be a valuable tool for elucidating the molecular mechanisms underlying the effects of factors associated with disease progression at the whole animal level. This would provide new insights into the pathogenesis of feline CKD, potentially informing future development of novel treatments for the management of veterinary cases and providing insight into the suitability of the cat as a model of human disease. Protocols for the isolation of human renal epithelial cells are well described [16], and several well-characterised immortalised cell lines also exist, including the proximal tubular cell line HK-2, which has been demonstrated to retain characteristics of freshly isolated proximal tubular cells [17]. However, there are currently no well-characterised feline tubular epithelial cell lines available, or published studies describing primary renal cell cultures from feline tissue.

The cytokine TGF-β1 is thought to be the most important mediator driving the progression of interstitial fibrosis in CKD, with upregulation of TGF-β1-driven signalling resulting in a host of pro-fibrotic effects which have led to its description as the “master regulator of fibrosis” [18]. TGF-β1 stimulates the transcription of genes related to ECM production in multiple cell types, both directly and indirectly via downstream pro-fibrotic mediators, such as connective tissue growth factor (CTGF) [19]. *In vitro*, tubular epithelial cells acquire a mesenchymal phenotype in response to TGF-β1 stimulation, via “epithelial to mesenchymal transition” (EMT), as indicated by the loss of epithelial markers, such as E-cadherin and cytokeratin, and gain of mesenchymal markers, such as N-cadherin, vimentin and genes related to ECM production [20]. This phenotype is thought to represent a maladaptive response to injury which contributes to the progression of renal fibrosis [21]. TGF-β1 is also a potent inhibitor of epithelial cell proliferation [22], and, at high concentrations, has also been reported to cause apoptosis [23]. Increased urinary TGF-β1 excretion is associated with interstitial fibrosis in cats, suggesting that this cytokine may play a causative role in the development of fibrosis, as in other species [24]. However, there are currently no studies examining the effects of TGF-β1 on feline renal cells, and whether this cytokine has analogous effects at a cellular level is unknown.

In the present study, we isolated proximal tubular epithelial cells (FPTEC) from the cadaverous kidney tissue of cats with normal kidney function, and characterised these cells in comparison to primary human renal epithelial cells (HREC) and the human proximal tubular cell line HK-2. We then compared the effects of human recombinant TGF-β1 on cell proliferation, pro-apoptotic signalling and genes associated with EMT in FPTEC, HREC and HK-2.

## Materials and methods

### Isolation and culture of feline proximal tubular cells (FPTEC), HREC and HK-2

Kidneys (and other tissues) were obtained from cats euthanased for welfare reasons (not related to kidney disease), with owner informed consent. The project protocol, owner information sheet and consent forms were approved by the RVC’s Ethics and Welfare Committee (URN 2013 1258, 2/12/2013). A previously published method for the isolation and culture of human renal proximal tubular epithelial cells was modified for use in cats [25]. Briefly, the renal cortex was minced and dissociated in DMEM/F12 containing 1mg/ml collagenase (collagenase A from *clostridium hemolyticum*, Roche) for 25 min at 37°C in a water bath. The digested tissue was centrifuged for 3 min at 90 x g, resuspended in DMEM/F12 and passed through a 212-μm sieve set on top of a 106-μm sieve into a 100mm tissue culture dish. The sieved cells were centrifuged for 3 min at 90 x *g* and resuspended in 10ml primary culture medium. Primary culture medium consisted of DMEM/F12, no phenol red, containing 2.5 mM L-glutamine and 15mM HEPES (Thermofisher scientific) supplemented with 10 ng/ml epidermal growth factor (Invitrogen), 36 ng/ml dexamethasone (Sigma-Aldrich), 2 ng/ml triiodothyronine (Sigma-Aldrich), 1% insulin-transferrin-selenium (Thermofisher scientific) and 1% antibiotic-antimycotic (Thermofisher scientific). The resuspended mixture was left to settle for 10 min, and the supernatant discarded. The remaining pellet was resuspended in primary culture medium and transferred into a 75cm^2^ tissue culture flask (Nunclon^™^ delta surface). Cells were maintained at 37°C in a 5% CO_2_/95% air humidified incubator (BB15 CO_2_; Thermofisher scientific). After 24 h the medium was removed and the cells washed with Dulbecco’s phosphate buffered saline (DPBS; Life technologies) before replacement of fresh primary culture medium. Thereafter, primary culture medium was replaced every 48 h. Once 75–80% confluent, the cells were trypsinized and distributed into tissue culture flasks/plates as appropriate at a 1:3 subculture ratio. All experiments on FPTEC were performed at passage 1 or 2.

Human renal epithelial cells (HREC) were purchased at passage 2 from Promocell (C-12665) and cultured according to the manufacturer’s instructions. Cells were defrosted in a 37°C water bath and seeded into a 75cm^2^ tissue culture flask containing proprietary renal epithelial cell growth medium (C-26030, Promocell) and supplement mix (C-39606, Promocell). Medium was replaced every 48 h and cells were subcultured at a 1:3 ratio once 80% confluent. All experiments were performed on cells at passage 2 or 3. Cryopreserved HK-2 cells (CRL-2190, ATCC) were purchased and cultured according to the manufacturer’s protocol. HK-2 were defrosted in a 37°C water bath and seeded into a 75cm^2^ tissue culture flask containing DMEM/F12 supplemented with 10% FBS. HK-2 cells were fed every 72 h and subcultured at a 1:4 ratio. Human umbilical cord collection (obtained with informed written consent) conformed to the principles outlined in the Declaration of Helsinki and is approved by the NHS Health Research Authority East of England-Cambridge South Research Ethics Committee (REC reference 16/EE/0396). Human umbilical vein endothelial cells were isolated and cultured as described previously and were used at passage 2 [26, 27].

Images of cells were collected using a DMIRB inverted microscope with samples illuminated using an EBQ100 light source (Leica Microsystems, Milton Keynes, UK) and an AxioCam ICm1 monochrome camera controlled through Axiovision software version 4.8.2 (Carl Zeiss Ltd, Cambridge, UK).

### Immunofluorescent detection of proteins in cultured cells

Cells were assessed for the expression of the marker proteins cytokeratin AE1/AE3, vimentin, desmin, α-klotho and von Willebrand factor (vWF) (FPTEC and HK-2 only) by immunofluorescence. Cells were plated onto collagen I-coated glass bottomed chamber slides (Nunc Lab-tek II chamber slide^™^, Sigma-Aldrich), left to adhere, then fixed in 4% formaldehyde for 15 min. Fixed cells were incubated with 50mM ammonium chloride (Sigma-Aldrich) for 15 min, washed three times with tris-buffered saline (TBS, pH 7.4) and permeabilized with 0.1% triton in TBS. Cells were incubated in blocking buffer (3% BSA, 1% goat serum, 0.1% tween in TBS) for 2 h at room temperature, then incubated with the desired primary antibody diluted in blocking buffer for 1 h at room temperature. Details of the primary antibodies used are provided in S1 Table. After three washes with TBS, labelled cells were incubated with the respective fluorescent secondary antibody (1:1000 in blocking buffer) for 1 h in the dark. After three further washes with TBS, the chambers were removed and a coverslip mounted using Fluoroshield^™^ with DAPI (Sigma-Aldrich). Images were collected using a DM4000B upright microscope with samples illuminated using an EBQ100 light source and filter cubes A4, L5 and TX2 (all from Leica Microsystems, Milton Keynes, UK) and an AxioCam MRm monochrome camera controlled through Axiovision software version 4.8.2 (Carl Zeiss Ltd, Cambridge, UK). Each experimental repeat included an isotype control matched to the species and isotype of the primary antibody used. Experiments were carried out in triplicate.

### Immunofluorescent detection of proteins in feline tissue

The distribution of the previously listed marker proteins was examined in formalin-fixed paraffin-embedded feline kidney tissue by immunohistochemistry. Sections (5 μm) were mounted, deparaffinized, and rehydrated via immersion in graded ethanol dilutions. Heat-induced epitope retrieval was performed in pH 9 Tris-EDTA buffer at 95°C for 25 min. Tissue sections were permeabilized with 2 x 5 min incubations in TBS + 0.025% Triton X-100 (Sigma-Aldrich) then incubated in blocking buffer (3% BSA, 1% goat serum, 0.1% tween in TBS) for 2 h at room temperature. The sections were then incubated with primary antibody (diluted in blocking buffer) overnight at 4°C (S1 Table**)**. The sections were subsequently washed twice in TBS pH 7.4 + 0.025% Triton X-100 and the fluorophore-conjugated goat secondary antibody applied at a dilution of 1:1000 in blocking buffer. Sections were incubated for 1 h in the dark at room temperature, before washing three times for 5 min each in TBS (pH 7.4). Autofluorescence was blocked prior to microscopy by incubating sections in 0.2% Sudan Black B (SBB; Sigma-Aldrich), diluted in 70% ethanol, for 20 min. Three washes of 5 min each in TBS (pH 7.4) and 0.02% Tween 20 (Sigma-Aldrich) were required for complete removal of excess SBB. A coverslip was then mounted using Fluoroshield^™^ with DAPI (Sigma-Aldrich) and images were collected using a DM4000B upright microscope. Negative controls were performed in parallel for every tissue section stained using a commercially available immunoglobulin isotype control matched to the species and isotype of the primary antibody. Experiments were repeated three times, where each repeat represented tissue sourced from a different cat.

### Western blotting

Western blotting was carried out as described previously [27]. Confluent monolayers of FPTEC/HK-2 treated as indicated were washed with ice cold DPBS containing 0.4 mM sodium orthovanadate (Sigma-Aldrich), before lysis in 68.3 mM Tris-HCl (Sigma-Aldrich) containing 10% (w/v) glycerol (Sigma-Aldrich), 2% (w/v) sodium dodecyl sulphate (SDS; Sigma-Aldrich), 2 mM sodium orthovanadate and 10 μL/ml protease inhibitor cocktail (Sigma-Aldrich). Proteins (25 μg) were separated by SDS polyacrylamide gel electrophoresis (SDS-PAGE) using pre-cast gels (10% Mini-protean TGX Stain-Free^™^ protein gel, Bio-rad), before transfer onto a polyvinylidene difluoride (PVDF) membrane (0.45 μm pore, Hybond^™^; GE Healthcare). Membranes were blocked for 3 h at room temperature in 5% (w/v) milk (Marvel) made up in Tris-buffered saline with Tween (TBST; 50 mM Tris, 150mM NaCl, 0.02% (v/v) Tween 20, pH 7.4). For immunodetection of proteins of interest, membranes were incubated overnight at 4 °C with primary antibody diluted in TBST + 10% (w/v) BSA containing anti-vimentin (1:1000), anti-cytokeratin AE1/AE3 (1:1000), anti-vWF (1:1000), or anti-β-actin (1:5000) antibody. Subsequent to this, membranes were washed in TBST (6 x 10 min) and then incubated with horse radish peroxidase (HRP)-conjugated goat anti-rabbit/mouse IgG as appropriate (1:10,000) in TBST containing 0.2% (w/v) BSA for 1 h at room temperature. After further washing (6 x 10 min), immunoreactive bands were detected via enhanced chemiluminescence (ECL). This was performed by exposing the blots to 0.1 M Tris pH 8.5 containing 0.25mg/ml luminol (Sigma-Aldrich), 0.18mg/ml 4-iodophenol (Sigma-Aldrich), 0.01% (v/v) H_2_O_2_ (Sigma-Aldrich) for 1 min, and visualising bands using photographic film (Hyperfilm^™^, Kodak). Experiments were carried out in triplicate. HUVEC protein lysates were used as positive controls for expression of the endothelial marker vWF.

### Enzyme cytochemistry

#### Gamma glutamyltranspeptidase (GGT)

FPTEC and HK-2 were stained for GGT expression using a modified version of a previously published protocol [28]. Cells were grown in 12 well culture plates (Nunclon^™^ delta surface) and shock-frozen in hexane (Sigma-Aldrich) for 5 min. The cells were then incubated for 45 min in a solution of 400 μM γ-glutamyl-4-methoxy-2-naphthylamide **(G**MNA), 30 μM glycylglycine, 1.2 mM Fast Blue BBN (Sigma Aldrich), and 0.2 M (pH 7.4) TBS. Cells were rinsed in 0.85% NaCl before a second incubation in 0.1 M CuSO_4_ (Sigma-Aldrich) for 2 min, then finally rinsed in distilled water. Images were collected using an Axiovert 135 inverted microscope (Carl Zeiss Ltd, Cambridge, UK) and an Infinity 3-3UR colour camera (Lumenera, Ottawa, ON, Canada) controlled through Image Pro Insight software version 9.1.4 (Media Cybernetics, Rockville, MD, USA). Experiments were carried out in triplicate.

#### Alkaline phosphatase (ALP)

FPTEC and HK-2 were stained for ALP expression using a commercially available ALP substrate solution (SIGMAFAST^™^ BCIP^®^/NBT, Sigma Aldrich). Cells grown in 12 well culture plates were fixed in 4% formaldehyde for 60 seconds, washed in phosphate buffered saline (PBS, Sigma-Aldrich), then incubated with the BCIP/NBT solution (1 ml) in the dark for 30 min. Images were collected using the Axiovert 135 inverted microscope. Experiments were carried out in triplicate.

### RT-qPCR analysis of gene expression

RNA was extracted from cells treated as indicated using a column based kit (Genelute^™^ Mammalian Total RNA Miniprep Kit, Sigma-Aldrich). Messenger RNA (mRNA) templates were reverse transcribed to complementary DNA (cDNA) using a commercially available kit (Omniscript RT, Qiagen), oligo dT primer (MWG Eurofins) and 1 U/ml RNaseOUT (Life Tehnologies). Gene expression was quantified by RT-qPCR in 96-well plates (Framestar^™^, Fortitude) using a commercially available SYBR Green *Taq* ready mix (SYBR^®^ Green JumpStart^™^ Taq ReadyMix^™^, Sigma-Aldrich), and was performed in a CFX Connect^™^ Real-Time PCR Detection System (Bio-Rad).

Collagen type 1α1 (Col1α1), fibronectin, alpha smooth muscle actin (α-SMA), transglutaminase 2 (TG-2), CTGF and TGF-β1 gene expression was analysed by RT-qPCR and normalised to GAPDH/RPS7 using primers listed in S2 Table. Experiments on FPTEC were performed in quadruplicate, where each experimental repeat represented a separate isolation from an individual cat. Experiments on HREC and HK-2 cells were performed in triplicate, with each experimental repeat representing a separate batch of cells. Data are expressed as mean fold change relative to untreated control and statistical significance was evaluated by one-way analysis of variance (ANOVA) with post-hoc Dunnet’s test. Statistical analyses were performed using GraphPad Prism software version 6.0 (Graphpad Software, La Jolla, CA).

### Measurement of cell proliferation

Cell proliferation was measured by counting cells stained with DAPI after treatment as indicated for 72 h. Cell proliferation assays were performed on cells seeded at 5000 cells/well into 96-well cell culture plates (Nunc), with 6 technical replicates for each treatment. After 72 h cells were washed with DPBS before fixation in 4% formaldehyde for 15 min. Cells were then washed with DPBS before incubation with 1 μg/ml of DAPI diluted in DPBS for 10 min in the dark, followed by a final wash in DPBS. Low power images (2.5x) of the wells were collected using the DM4000B upright microscope. The number of cells per image was counted using freely available software (ImageJ). Experiments on FPTEC were performed in quadruplicate, where each experimental repeat represented a separate isolation from an individual cat. Experiments on the HREC and HK-2 cells were performed in triplicate, where each experimental repeat represented a separate batch of cells. Data are presented as fold change in cell number in comparison to control.

### Measurement of caspase-3/7 activity

Caspase 3/7 activity was measured as an indicator of apoptotic activity in FPTEC and HK-2 cells using the Caspase-Glo^®^ 3/7 assay (Promega), as per the manufacturer’s instructions. This assay was performed on cells seeded into white-walled 96-well plates and cultured to confluence. Experiments on FPTEC were performed in quadruplicate, where each experimental repeat represented a separate isolation from an individual cat. Experiments on the HK-2 cells were performed in triplicate, where each experimental repeat represented a separate batch of cells. Results are presented as fold change in comparison to experimental control.

## Results

### Characteristics of FPTEC, HREC and HK-2 in culture

**Fig 1:**
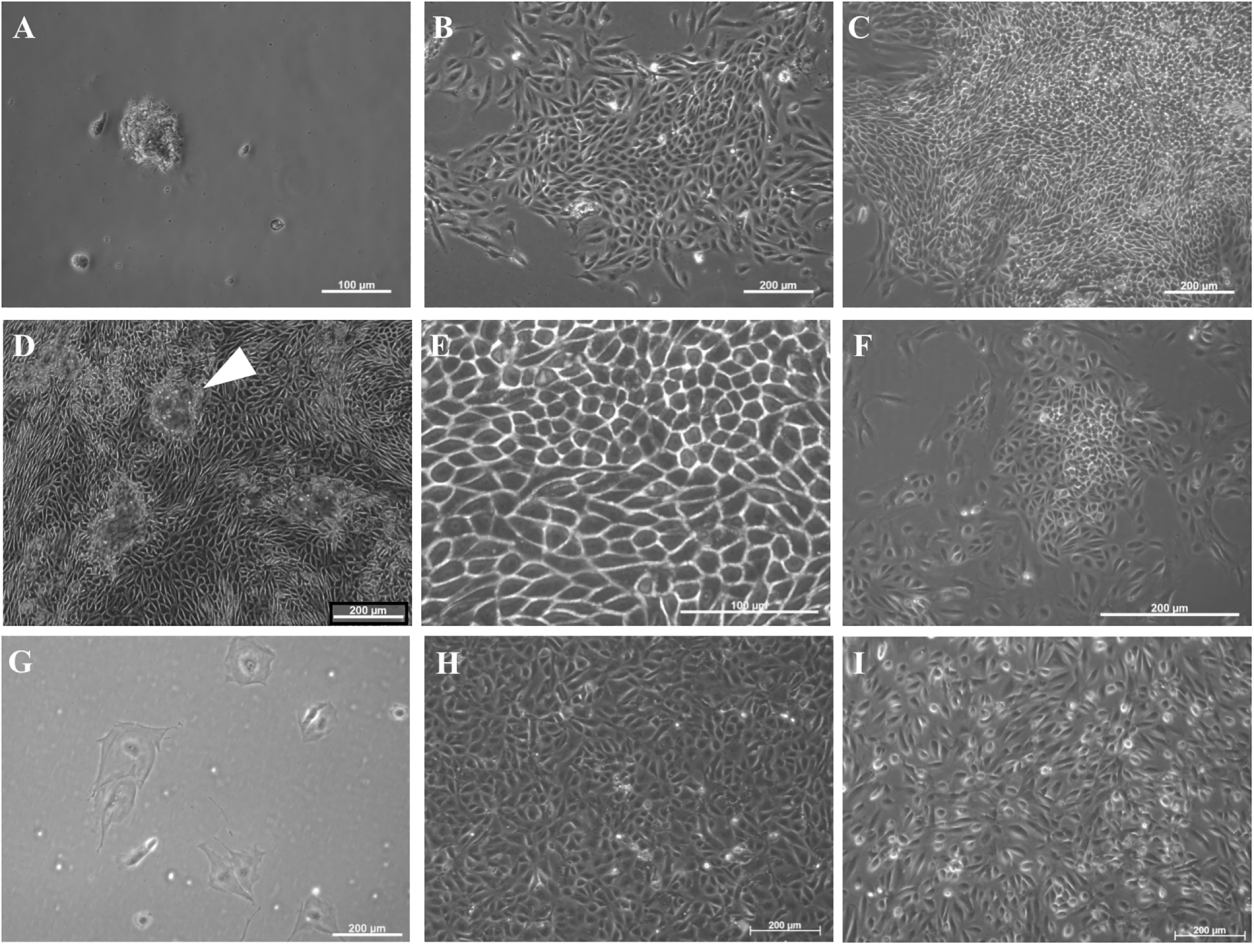
Morphology of FPTEC, HREC and HK-2. Representative photomicrographs of FPTEC, HREC and HK-2 cells. (A) FPTEC 24hrs post isolation. Small tissue clumps and individual cells are adhered to the tissue culture plastic. (B) FPTEC 96 hours post isolation. Proliferating colonies of epithelial cells form from the isolated tissue. (C) FPTEC 7 days post isolation. Colonies mature into densely packed epithelial monolayers. (D) FPTEC, passage 1. Cells maintain their epithelial morphology and demonstrate formation of “domes” (thick arrow) at confluency. (E) FPTEC, passage 1: High power image illustrating cuboidal morphology and close cell-cell adhesion. (F) FPTEC, passage 3. Larger, irregular cells begin to appear, indicating cultures are developing replicative senescence. (G) FPTEC, passage 4. The few remaining adherent cells are greatly enlarged and irregular. (H) HK-2 cells. The HK-2 cell line formed monolayers of cuboidal, epithelial cells and did not form domes. (I) HREC, passage 2. These cultures were initially heterogeneous, grew into a cuboidal monolayer. Images are representative of three experiments.

Cultures of FPTEC were successfully established from the kidney tissue of six cats with normal kidney function. Cultures were initially heterogenous but, after 7–10 days, became dominated by relatively homogenous monolayers of cells with a cuboidal, “cobblestone” morphology, which demonstrated contact inhibition and dome formation, characteristic of cultured kidney epithelial cells from other species [29, 30] (Fig 1A–1E). The cells maintained an epithelial phenotype until passage three, when enlarged, irregular senescent cells began to appear, and at passage four all proliferative capacity was lost (Fig 1F and 1G). HREC cultures were initially heterogeneous before forming a cuboidal monolayer which was maintained over the range of guaranteed population doublings (Fig 1I). Dome formation was not observed. HK-2 cells formed monolayers of cuboidal, contact-inhibited cells, and dome formation was not evident (Fig 1H). Cells grew rapidly, and have been cultured successfully for over 10 passages with no loss of morphology or proliferative capacity.

### Marker protein expression in FPTEC, HREC, HK-2 and normal feline tissue

**Fig 2:**
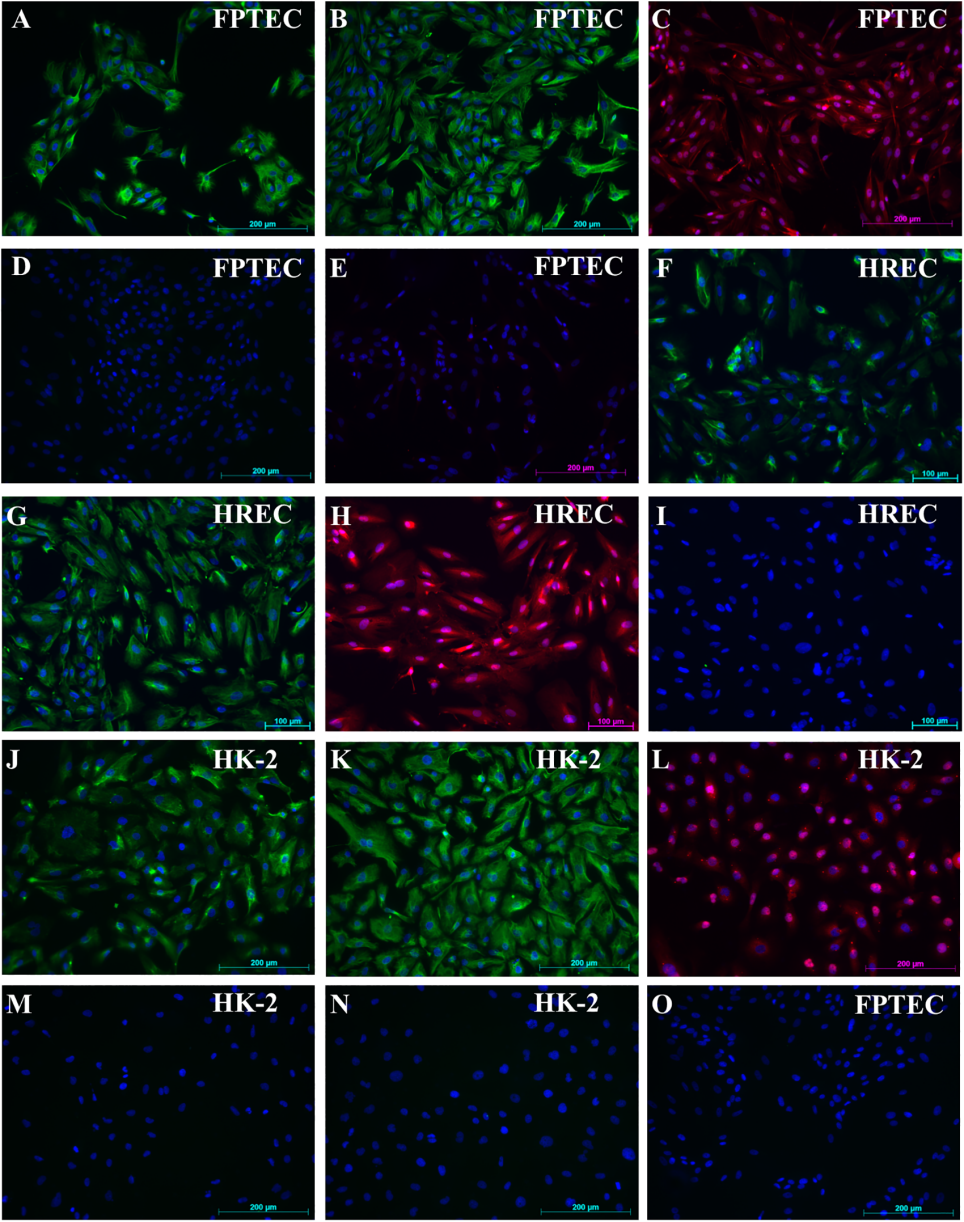
Immunofluorescence studies of FPTEC, HREC and HK-2. Immunofluorescence staining of FPTEC, HREC and HK-2. Cell nuclei were stained with DAPI (blue). FPTEC were (A) >85% positive for cytokeratin AE1/AE3 expression (green), (B) 100% positive for vimentin expression (green), (C) α-klotho expression (red), and negative for (D) desmin and (E) vWF. HREC were 100% positive for (F) cytokeratin (green), (G) vimentin (green), (H) α-klotho (red) and negative for (I) desmin. Similarly, HK-2 were 100% positive for (J) cytokeratin (green), (K) vimentin (green), (L) α-klotho (red) and negative for (M) desmin and (N) vWF. (O) Isotype controls were negative. Images are representative of three experiments.

The cytokeratin AE1/AE3 antibody labelled distal tubular epithelia and collecting duct epithelia in the normal feline kidney (S1 Fig). By immunofluorescence, HREC and HK-2 cells stained uniformly positive for cytokeratin, as did >85% of the FPTEC (Fig 2A, 2F and 2J), and expression was confirmed by western blotting (S2 Fig). Glomeruli, parietal epithelial cells, scattered interstitial cells and some endothelial cells expressed vimentin in the normal feline kidney (S1 Fig). HREC, HK-2 and FPTEC were uniformly positive for vimentin expression by immunocytochemistry (Fig 2B, 2G and 2K) and western blotting (S2 Fig) (HK-2 and FPTEC only). Desmin immunofluorescence could not be detected in the normal feline kidney, but strong expression was demonstrated in feline bladder smooth and striated muscle (S1 Fig). HREC, HK-2 and FPTEC were negative for desmin expression by immunocytochemistry (Fig 2D, 2I and 2M). In line with this observation, desmin could not be detected by immunoblotting.

**Fig 3:**
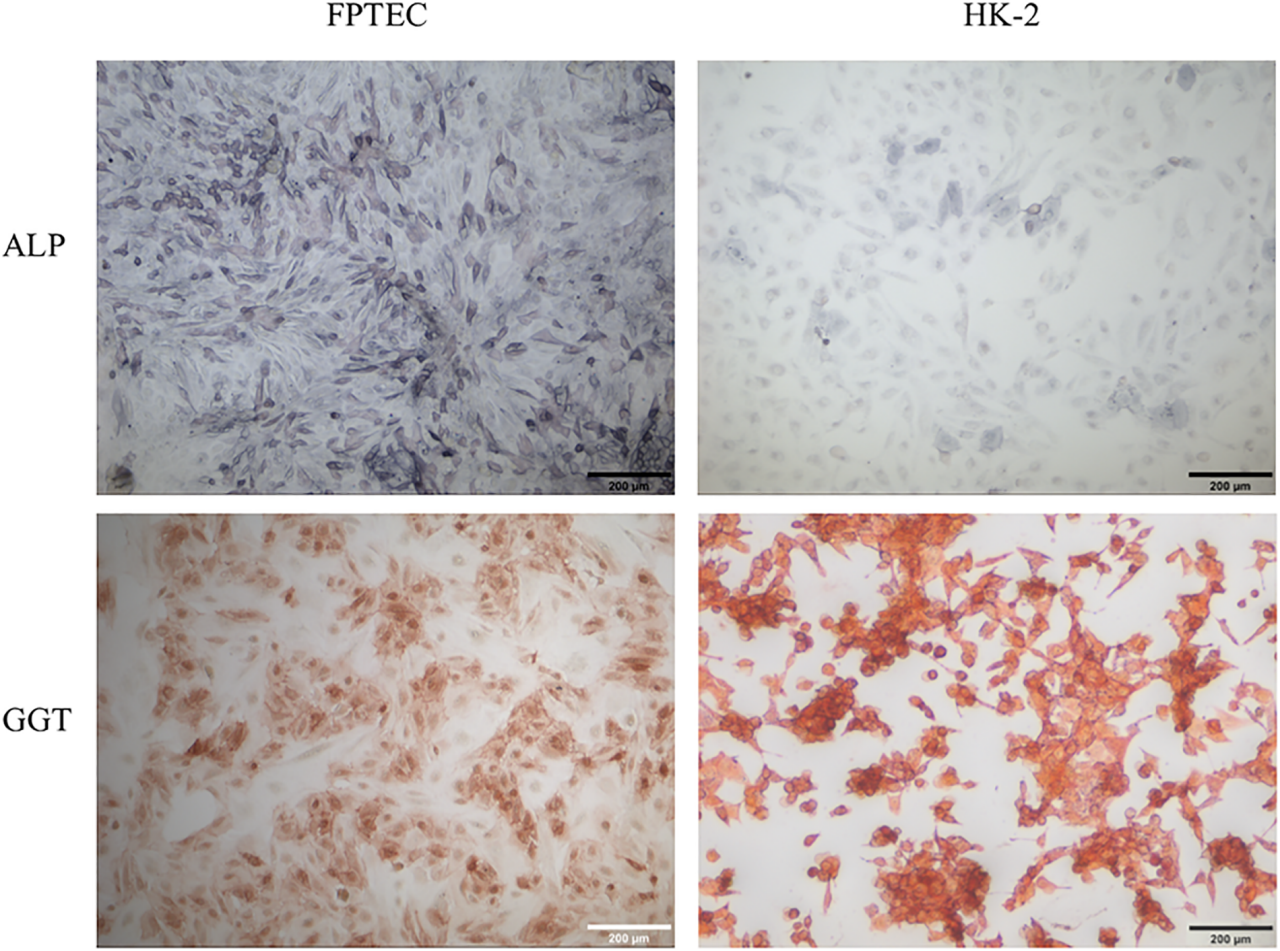
Brush border enzyme expression in FPTEC and HK-2. Photomicrographs of cells stained for the demonstration of ALP (blue/mauve) and GGT (red/orange) activities. FPTEC stained positive for both ALP and GGT activity. Expression was heterogenous in both cases and ranged from intense to barely present. HK-2 demonstrated lower ALP activity, with staining generally less intense than the FPTEC, with some heterogenicity, but stained intensely and homogeneously positive for GGT expression. Images are representative of three experiments.

Membrane bound α-Klotho is as an obligate co-receptor for the phosphaturic hormone FGF-23, and can be regarded as a tubular specific marker protein [31, 32]. In the normal feline kidney, there was strong expression of α-Klotho in distal tubules and moderate expression in proximal tubules (S1 Fig). HREC, HK-2 and FPTEC were uniformly positive for α-Klotho by immunocytochemistry (Fig 2C, 2H and 2L) and western blotting (S2 Fig**)** (HK-2 and FPTEC only). The vWF antibody labelled interstitial endothelial cells in the normal feline kidney (S1 Fig), and both HK-2 and FPTEC were negative for vWF expression by immunofluorescence (Fig 2E and 2N) and western blotting (S2 Fig). HK-2 cells stained weakly positive for ALP expression, and strongly positive for GGT expression by enzyme cytochemistry (Fig 3). In the FPTEC cultures, ALP staining was stronger and GGT staining weaker, with more heterogeneity in expression of both enzymes.

### TGF-β1 induces Smad signalling and changes in gene expression consistent with EMT in FPTEC, HREC and HK-2

**Fig 4:**
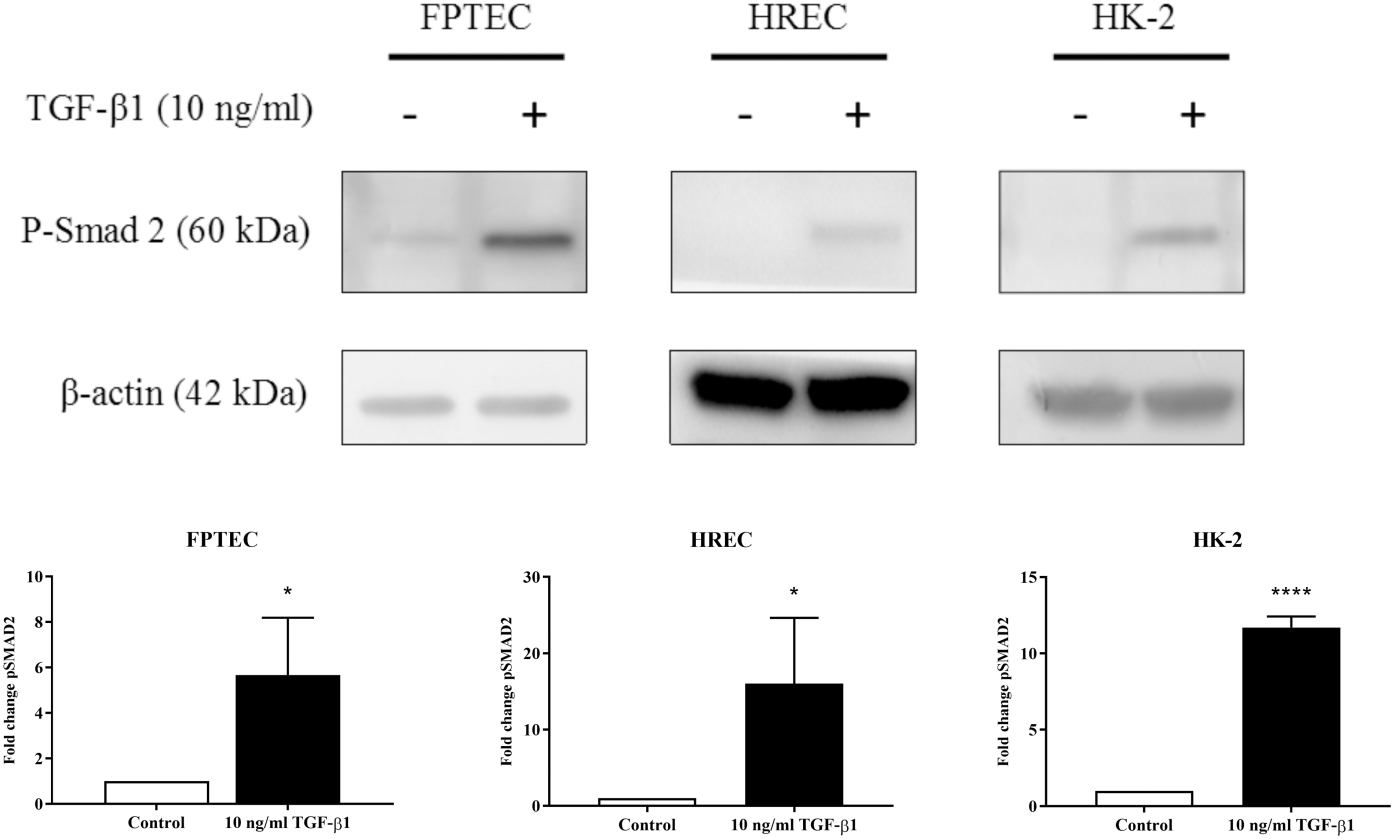
Effect of TGF-β1 on phosphorylation of Smad2 in FPTEC, HREC and HK-2. The effect of a 30 minute treatment with 10ng/ml TGF-β1 on the expression of phosphorylated Smad2 (p-Smad2) in FPTEC, HREC and HK-2 was assessed by western blotting. Densitometric analysis of p-Smad2 was undertaken in ImageJ, normalised to β-actin and is expressed as fold change in relation to control. Incubation with TGF-β1 resulted in a significant increase in p-Smad2 expression in FPTEC (P = 0.038), HREC (*P* = 0.0393) and HK-2 (P < 0.0001). Data were analysed using the Student’s t-test. The columns represent the mean normalised density measurement and error bars represent the standard deviation. Images are representative of three experiments. *P<0.05, ****P<0.0001.

**Fig 5:**
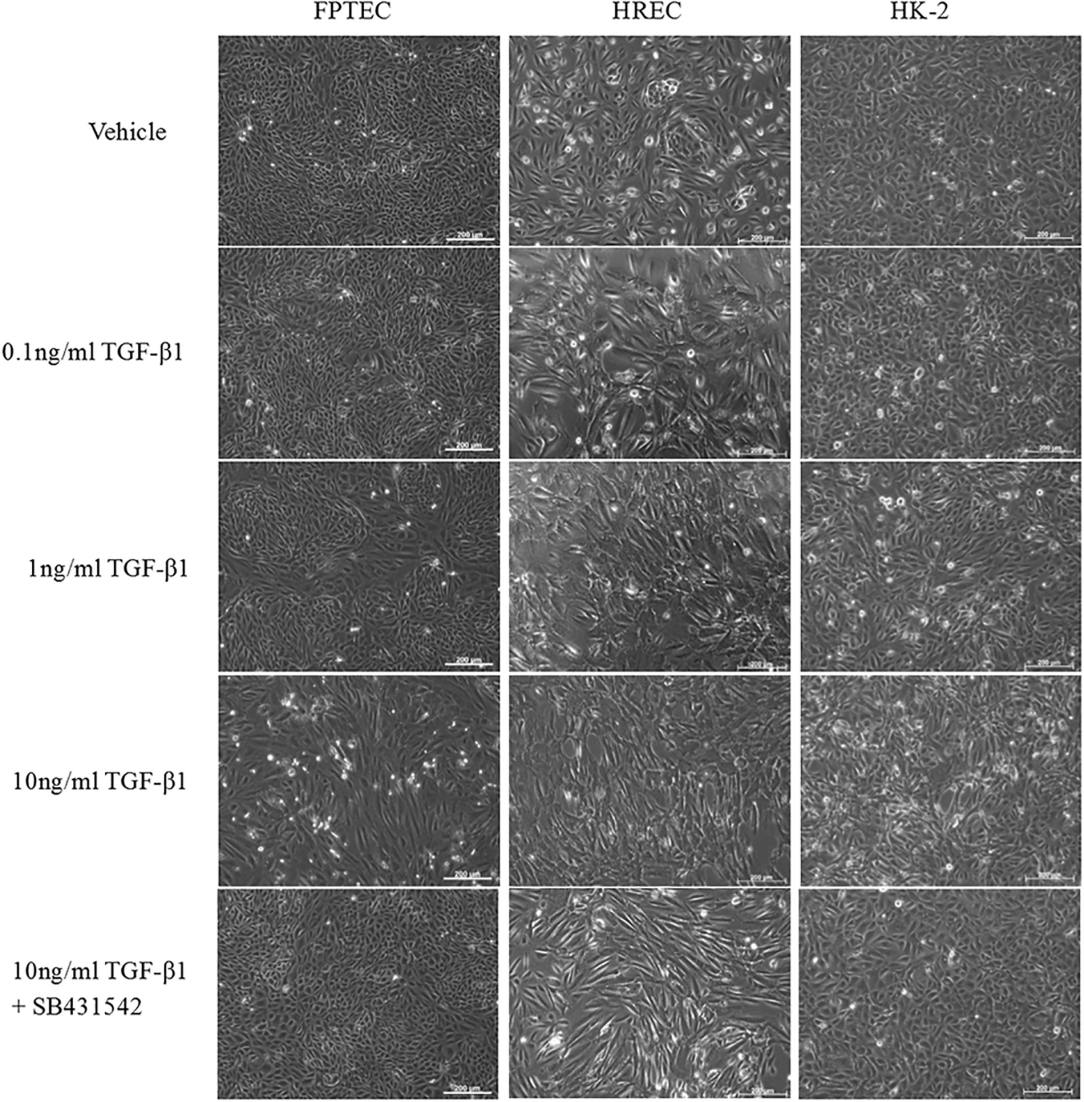
Effect of TGF-β1 on FPTEC, HREC and HK-2 cell morphology. Representative photomicrographs of FPTEC, HREC and HK-2 after incubation with vehicle and 0.1–10 ng/ml TGF-β1 for 72 h. There was a concentration-dependent loss of the morphology evident in vehicle-treated cells across all three cell types, with cells adopting a hypertrophic, elongated fusiform appearance. These alterations were inhibited by the TGF-β1R1/ALK5 antagonist SB431542 (5 μM). Images are representative of three experiments.

Treatment with 10 ng/ml TGF-β1 for 30 min resulted in a significant increase in Smad2 phosphorylation in FPTEC (*P* = 0.038), HREC (*P* = 0.039) and HK-2 (*P* < 0.0001) (Fig 4). After 72 h treatment with TGF-β1, there was a concentration-dependent alteration in cell morphology consistent with EMT in all cell types (Fig 5). Cells became hypertrophied, elongated and spindle-shaped, with gaps in the monolayer where dead cells had detached at higher concentrations of TGF-β1. These changes were abrogated by treatment with the TGF-β1R1 antagonist SB431542 (5 μM) in all cell types.

**Fig 6:**
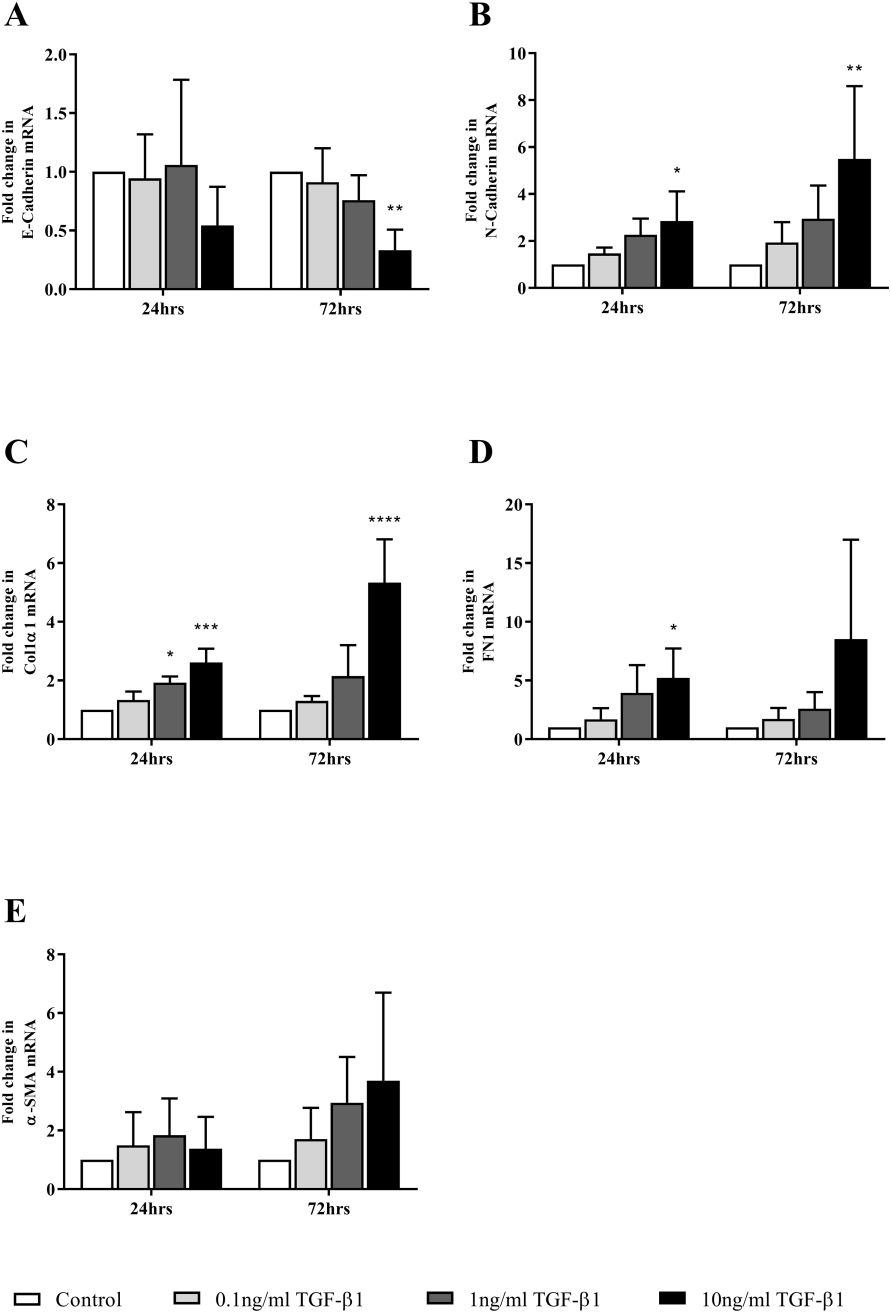
TGF-β1 mediated expression of genes related to EMT in FPTEC. Expression levels of commonly accepted EMT marker genes were assessed in FPTEC by RT-qPCR after incubation with TGF-β1 (0.1 to 10 ng/ml). Target gene mRNA copy number was normalised to GAPDH/RPS7 and is expressed as fold change in relation to control. Data represent four experimental repeats using cells from different cats and were analysed using the one-way ANOVA with Dunnett’s post-hoc analysis. The columns represent the mean normalised mRNA copy number and error bars represent the standard deviation.*P<0.05 **P<0.01 ***P<0.001 ****P<0.0001.

**Fig 7:**
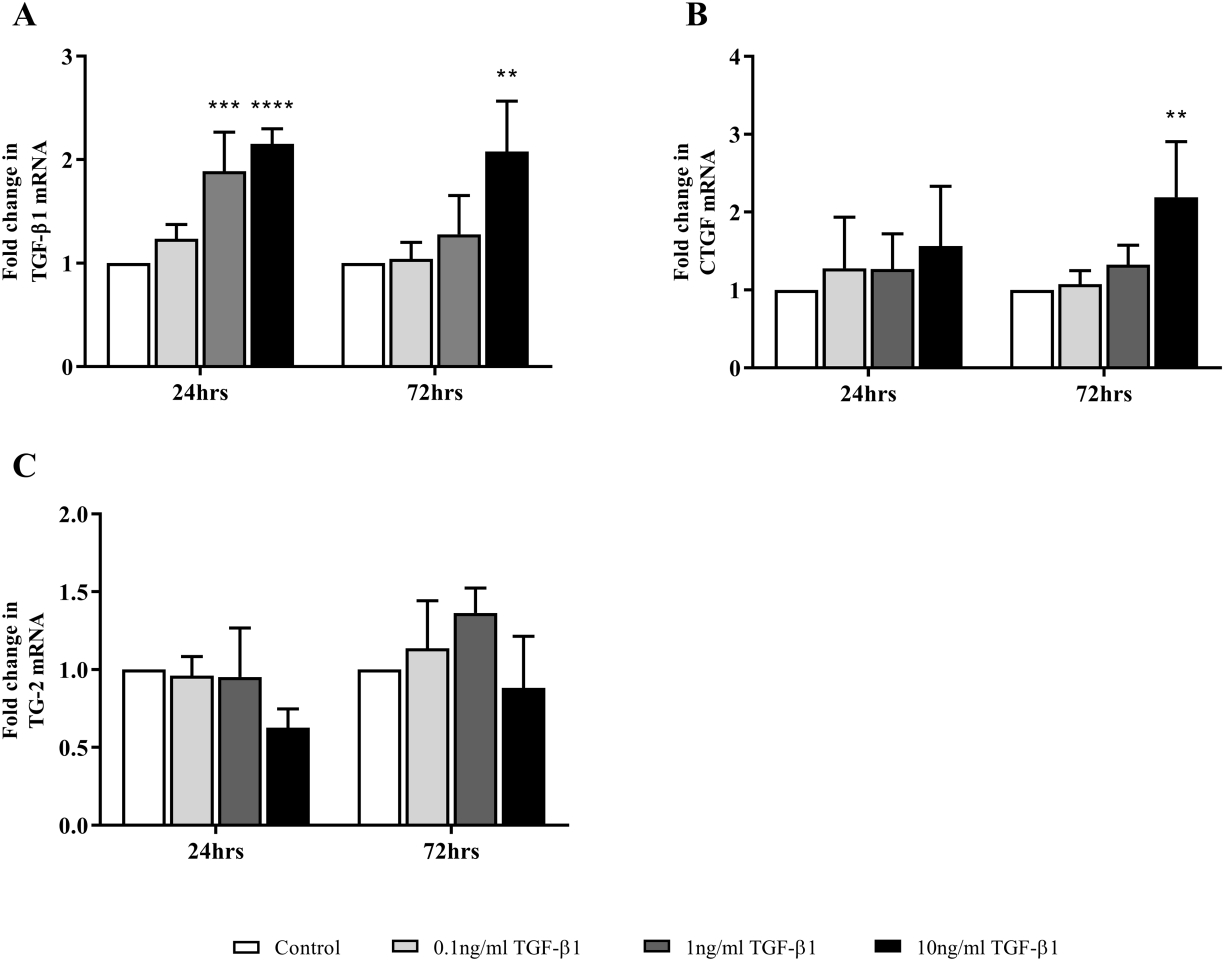
TGF-β1-mediated expression of downstream mediator mRNA in FPTEC. Expression of paracrine and autocrine mediator mRNA after incubation with TGF-β1 was assessed in FPTEC by RT-qPCR. Target gene mRNA copy number was normalised to GAPDH/RPS7 and is expressed as fold change in relation to control. Data represent four experimental repeats using cells from different cats and were analysed using the one-way ANOVA with Dunnett’s *post-hoc* analysis. The columns represent the mean normalised mRNA copy number and error bars represent the standard deviation. *P<0.05 **P<0.01 ***P<0.001 ****P<0.0001.

The expression level of a number of genes commonly associated with EMT was assessed in FPTEC by RT-qPCR after treatment with a range of TGF-β1 concentrations up to 10 ng/ml. E-cadherin was significantly downregulated after 72 h (10 ng/ml, *P* = 0.0013) (Fig 6A), whilst there was an increase in N-cadherin expression at both 24 h (10 ng/ml, *P* = 0.01) and 72 h (10 ng/ml, *P* = 0.0095) (Fig 6B). TGF-β1 upregulated collagen type 1α1 expression at both the 24 h (1ng/ml, *P* = 0.013; 10 ng/ml, *P* = 0.0004), and 72h time points (10ng/ml, *P* = 0.0001) (Fig 6C), and fibronectin expression at the 24 h time point (10 ng/ml, *P* = 0.015) (Fig 6D). There was no significant change in α-SMA expression (Fig 6E). Significant auto-induction of TGF-β1 mRNA expression was evident after 24 h (1ng/ml, *P* = 0.002; 10 ng/ml, *P* = 0.001) and 72 h (10 ng/ml, *P* = 0.0012) (Fig 7A). There was also a significant increase in CTGF mRNA expression after 72 h (10ng/ml, *P* = 0.0026) (Fig 7B**)**, but no significant change in TG-2 mRNA expression after 24 h (*P* = 0.11) or 72h (*P* = 0.16) (Fig 7C).

**Fig 8:**
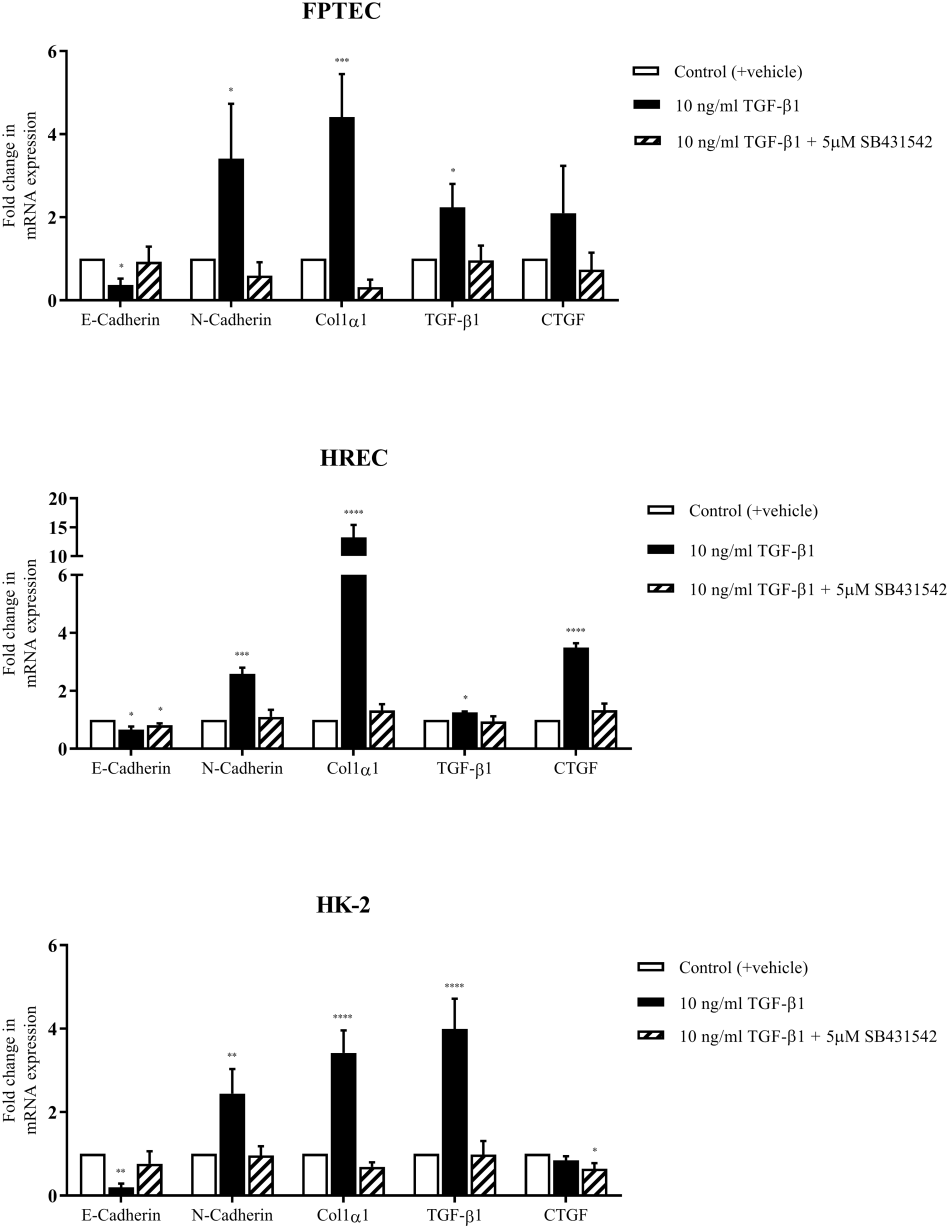
Comparative effect of TGF-β1 and the TGF-β1RI/ALK5 inhibitor SB431542 on target gene expression in FPTEC, HREC and HK-2. Effects of TGF-β1 (+/- 5 μM SB431542) on target gene expression was assessed in FPTEC, HREC and HK-2 by RT-qPCR. Target gene mRNA copy number was normalised to GAPDH/RPS7 and is expressed as fold change in relation to control. Data represent three experimental repeats using cells from different cats/batches and were analysed using the one-way ANOVA with Dunnett’s *post-hoc* analysis. The columns represent the mean normalised mRNA copy number and error bars represent the standard deviation.*P<0.05 **P<0.01 ***P<0.001.

Separate experiments were performed to compare the effect of TGF-β1 on a selected subset of genes in FPTEC, HREC and HK-2, and to evaluate the effects of SB431542 on TGF-β1-induced alterations in gene expression. In these experiments, 10 ng/ml TGF-β1 induced upregulation of collagen type 1α1 (*P* = 0.0008), N-cadherin (*P* = 0.017) and TGF-β1 (*P* = 0.013), and downregulation of E-cadherin (*P* = 0.026) in FPTEC (Fig 8). In the presence of 5 μM SB431542 these changes were inhibited, and gene expression was no different from control. CTGF mRNA expression was not significantly upregulated by TGF-β1 in FPTEC in this group of experiments, but trended towards significance (*P* = 0.11). These results were consistent with those of identical experiments performed in HREC, where 10 ng/ml TGF- β1 significantly upregulated expression of collagen type 1α1 (*P* < 0.0001), N-cadherin (*P* < 0.0001), TGF-β1 (*P* = 0.04) and CTGF (*P* < 0.0001), and downregulated E-cadherin expression (*P* = 0.0018) (Fig 8**)**. Treatment with SB431542 (5 μM) inhibited these alterations in gene expression, although E-cadherin expression was still significantly lower than control (*P* = 0.03). Finally, in HK-2 cells TGF-β1 (10 ng/ml) also induced upregulation of collagen type 1α1 (*P* = 0.0002), N-Cadherin (*P* = 0.005), TGF-β1 (*P* = 0.0004) and TG-2 (*P* = 0.023), and downregulated E-Cadherin (*P* = 0.028) (Fig 8). Treatment with SB431542 (5 μM) completely inhibited these TGF-β1-induced changes in gene expression. CTGF mRNA expression was not affected by incubation with TGF-β1 alone (*P* = 0.16) but expression was significantly decreased relative to control in the presence of TGF-β1 and SB431542 combined (*P* = 0.008).

### TGF-β1 inhibits proliferation of FPTEC and HREC but not HK-2 cells

**Fig 9:**
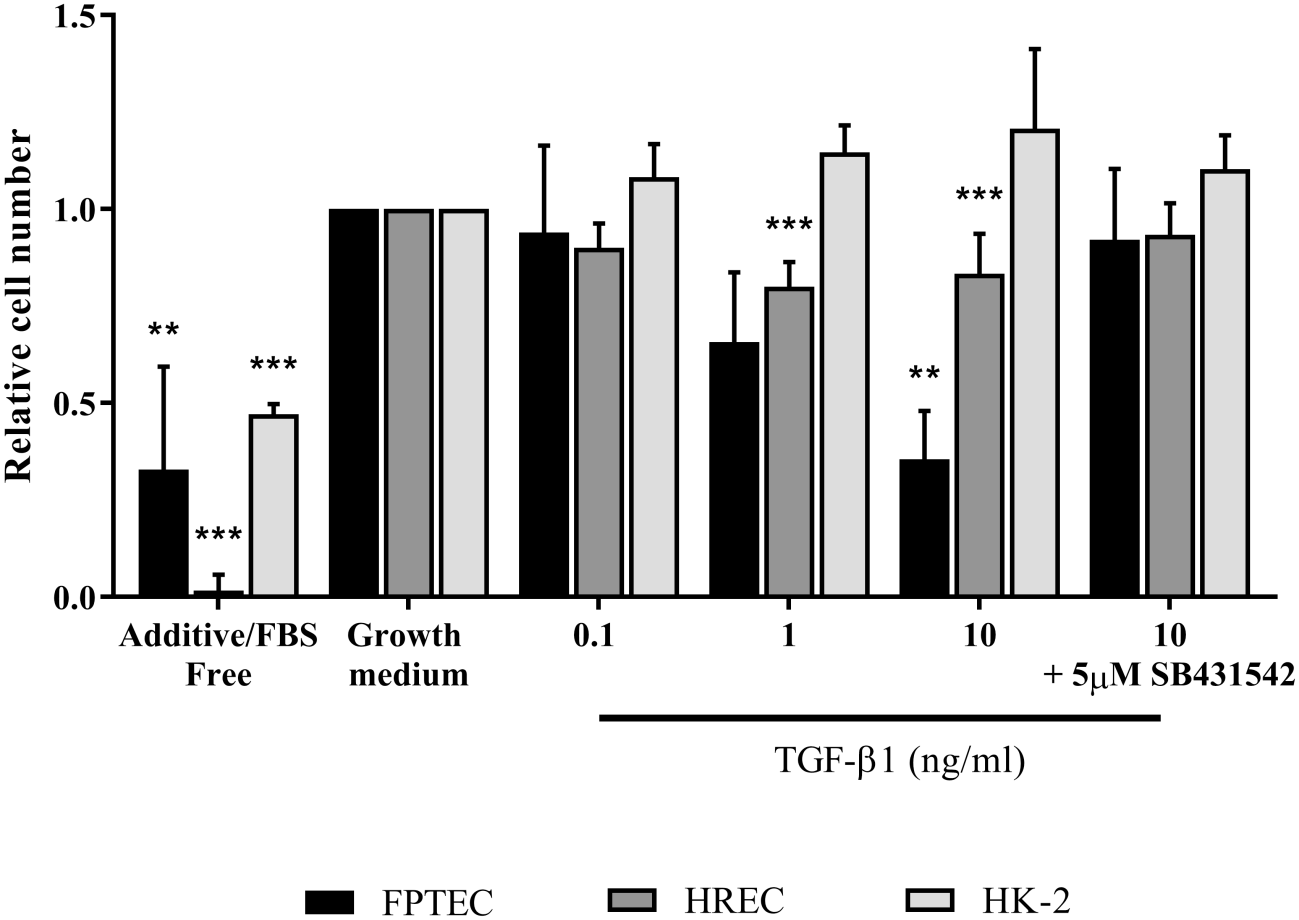
Effect of TGF-β1 on proliferation of FPTEC, HREC and HK-2. FPTEC, HREC and HK-2 were seeded onto 96-well plates at sub-confluent density and proliferation assessed by cell counting in DAPI-stained cultures after incubation with the indicated concentrations of TGF-β1 for 72 h. Data from FPTEC represent four experimental repeats using cells from different cats; data from HREC and HK-2 cells represent three experimental repeats. Data were analysed using the one way ANOVA with Dunnett’s post-hoc analysis. The columns represent the mean relative cell number and error bars represent the standard deviation.*P<0.05 **P<0.01 ***P<0.001.

We next investigated the effects of TGF-β1 on cell proliferation. There was a negative effect of TGF-β1 on the proliferation of FPTEC and HREC after 72 h, which appeared to be concentration-dependent (Fig 9). In the FPTEC experiments, there was a trend towards a significant decrease in cell number relative to control after incubation with 1 ng/ml TGF-β1 (*P* = 0.055), and a significant decrease with 10ng/ml TGF-β1 (*P* = 0.004). In the presence of 5 μM SB431542, the negative effect of 10 ng/ml TGF-β1 on cell proliferation was lost, and cell number did not differ significantly from control (*P* = 0.95). In the HREC experiments, there was a significant decrease in cell number relative to control after incubation with 1 ng/ml (*P* = 0.0005) and 10 ng/ml (*P* = 0.0041) TGF-β1. Again, the negative effect of 10 ng/ml TGF-β1 was absent in the presence of 5 μM SB431542, with cell number no different from control (*P* = 0.4966). Treatment with TGF-β1 did not significantly modulate HK-2 cell proliferation after 72 h (*P* = 0.29).

### TGF-β1 upregulates pro-apoptotic signalling in FPTEC and HK-2

**Fig 10:**
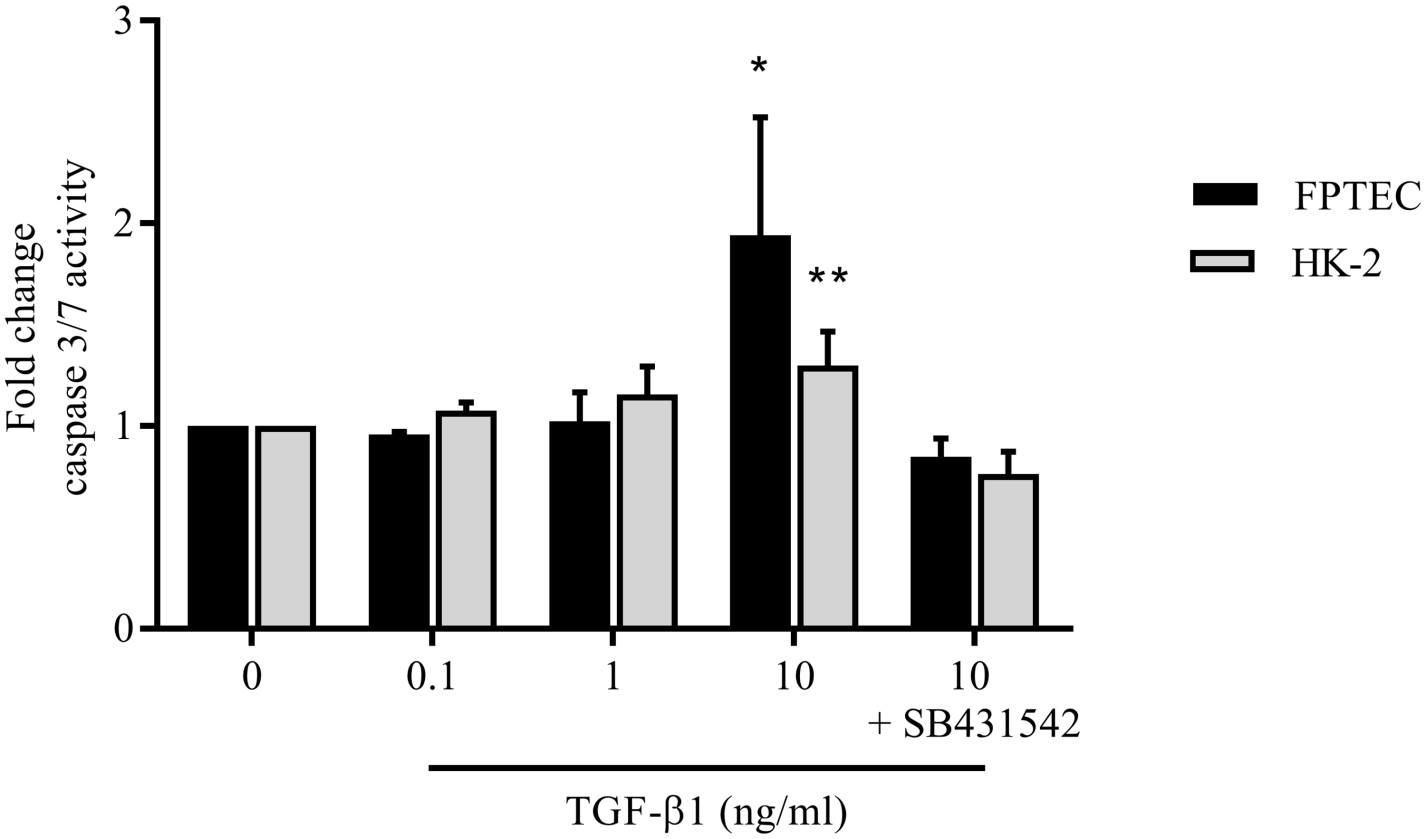
TGF-β1-induced changes in caspase 3/7 activity in FPTEC and HK-2. Pro-apoptotic signalling was assessed in HK-2 and FPTEC via measurement of caspase 3/7 activity. Data from FPTEC represent three experimental repeats using cells from different cats; data from HK-2 cells represent three experimental repeats. Data were analysed using the one way ANOVA with Dunnett’s *post-hoc* analysis. The columns represent the mean normalised mRNA copy number and error bars represent the standard deviation.*P<0.05 **P<0.01 ***P<0.*001*.

Pro-apoptotic signalling, as measured by caspase 3/7 activity, was significantly increased (by 30%) in HK-2 cells (*P =* 0.02) and by 90% in FPTEC (*P =* 0.006) after incubation with 10ng/ml TGF-β1 for 72 h (Fig 10), an effect that was inhibited by co-incubation with SB431542 (5 μM).

## Discussion

The first goal of the present study was to validate a method for the culture of feline PTEC, and these cells were characterised alongside primary HREC and HK-2—a human cell line which has been reported to retain characteristics of proximal tubular cells *in vitro* [17, 33]. The FPTEC demonstrated a marker protein expression profile identical to that of HREC and HK-2, namely positive for the epithelial intermediate filament cytokeratin, the tubular marker α-klotho and the mesenchymal marker vimentin, whilst negative for the myogenic marker desmin and endothelial marker vWF [34]. Both FPTEC and HK-2 were also positive for ALP and GGT activity, which is considered a specific feature of proximal tubular origin [17, 35–37]. The expression profile of all three renal epithelial cell types *in vitro* differed from proximal tubules *in situ*, as neither vimentin nor cytokeratin AE1/AE3 immunoreactivity were found in the proximal tubules of normal feline kidney sections, which is consistent with previous reports [38]. This phenomenon has been reported previously and is probably related to alterations in intermediate filament expression induced by cell culture conditions [34, 39]. In tubular epithelia altered by various injurious conditions, novel or enhanced expression of cytokeratin and vimentin is noted and may be a marker of proliferating tubular cells. It is therefore likely that the epithelial cells utilised in the present study represent an injured, somewhat de-differentiated proximal tubular phenotype, which is related to their ability to proliferate and is an unavoidable consequence of *in vitro* culture [40–43]. Importantly, however, cultures of FPTEC left at confluence formed domes, which is considered characteristic of renal tubular epithelial cells [30] and provides evidence that the FPTEC monolayer is maintaining the differentiated property of acting as a permeability barrier with apical and basolateral polarization *in vitro* [30]. Interestingly, HREC did not demonstrate dome formation in culture, which may indicate these cells are further de-differentiated in comparison to the FPTEC isolated in this study. HK-2 cells also did not form domes at confluence, an observation which has previously been reported, and may be due to a loss of certain transport proteins [44].

The canonical TGF-β1 signalling pathway involves TGF-β1 binding to the TGF-β1 type II receptor (TGF-β1RII), which recruits the TGF-β1 type I receptor (TGF-β1RI, also known as activin-like kinase 5 [ALK-5]) and forms a heteromeric receptor complex which phosphorylates receptor regulated Smad proteins (Smad2 and Smad3) [45]. These proteins subsequently regulate the transcription of target genes in the nucleus. In the present study, phosphorylated Smad2 expression was increased in FPTEC after TGF-β1 treatment, confirming that human recombinant TGF-β1 was able to initiate the intracellular Smad signalling pathway in feline FPTEC. This was consistent with results obtained utilising the HREC and HK-2 cells, and with previous literature on PTEC derived from other species [46, 47].

The TGF-β1/Smad signalling pathway is well characterised as central to the induction and perpetuation of fibrosis in naturally occurring human CKD, as well as experimental models of renal injury [18]. Recently, an association between urinary active TGF-β1 excretion and severity of renal interstitial fibrosis has also been reported in cats [24]. The present study is the first to show that increased extracellular concentrations of TGF-β1 induce pro-fibrotic alterations in feline FPTEC which are comparable to those documented in human cell types. Tubular epithelial cells exposed to TGF-β1, and other injurious factors, are believed to play a causal role in the development of tubulointerstitial fibrosis through paracrine cross-talk with the interstitium, resulting in myofibroblast activation and proliferation [48]. This pro-inflammatory, pro-fibrotic phenotype is associated with a loss of epithelial phenotype and a gain of mesenchymal markers through a process known as partial EMT or epithelial plasticity [21]. In the present study, cell morphology and gene expression in FPTEC, HREC and HK-2 were assessed as a measure of EMT in response to incubation with human recombinant TGF-β1. All three cell types exhibited an apparent concentration-dependent loss of epithelial morphology and development of a hypertrophic, fusiform phenotype in response to TGF-β1 stimulation. These morphological changes were accompanied by a decrease in the epithelial marker E-cadherin, and an increase in the mesenchymal markers N-cadherin, collagen type 1α1 and fibronectin. These changes are typical of EMT and are consistent with previous studies utilising HK-2 cells [49, 50] and human PTEC [46, 49, 51]. In the present study, there was no significant change in expression of α-SMA, a myofibroblast marker, in the FPTEC. Although α-SMA has been used previously as a marker of EMT [52], its suitability has been questioned by recent work suggesting that epithelial cells do not migrate into the interstitium and transdifferentiate into myofibroblasts [53].

The present study also identified that TGF-β1-induced EMT in both feline and human renal epithelial cells was accompanied by the upregulation of genes associated with paracrine signalling. A concentration-dependent auto-induction of TGF-β1 mRNA was apparent in all cell types, which is thought to be important in amplifying and sustaining effects of TGF-β1 [54]. The FPTEC also demonstrated a concentration-dependent induction of CTGF mRNA expression, which is a key downstream mediator of the pro-fibrotic effects of TGF-β1, particularly myofibroblast activation and ECM production [55, 56]. Whilst CTGF was induced by 10 ng/ml TGF-β1 in the HREC, this effect was not seen in the HK-2 cells. As CTGF expression has previously been established as responsive to TGF-β1 in HK-2 cells, as in other cell types [57], it was concluded that this was an aberrant result due to human error or environmental factors. Expression of TG-2, a cross-linking enzyme involved in stabilisation of the ECM and modulation of TGF-β1 signalling [58, 59], has been documented to be induced by TGF-β1 in previous studies utilising human PTEC [60, 61], however this did not appear to be the case in FPTEC in this study. It is unclear why this effect was not seen in FPTEC, but TGF-β1 does not induce TG-2 expression in all cell types *in vitro* [61], and it is possible that the upregulation of TG-2 in feline CKD occurs independently of the TGF-β1 signalling pathway. The small molecule TGF-β1RI/ALK5 antagonist SB431542 was found to inhibit TGF-β1-mediated effects on gene expression in FPTEC, HREC and HK-2, indicating that the effects of TGF-β1 on FPTEC are mediated by the TGF-β1RI(ALK5)/TGF-β1RII receptor complex in cats, as in other species [62, 63].

The TGF-β1 signalling pathway is well characterised as a negative regulator of epithelial cell proliferation [64]. This function is important in the context of fibrogenesis, as epithelial cell proliferation is essential for restoration of functional nephron integrity after renal injury, whereas epithelial cell growth cycle arrest is associated with a pro-fibrotic cellular phenotype [65, 66]. The present study revealed that TGF-β1 significantly inhibits FPTEC proliferation through activation of the TGF-β1RI(ALK5)/TGF-β1RII receptor complex. This was in accordance with results obtained using HREC and prior studies on PTEC derived from other species [67–69]. In contrast, there was no effect of TGF-β1 on the proliferation of HK-2 cells after 72 h. The HK-2 cell line has been shown in previous studies to be relatively resistant to the cytostatic effect of TGF-β1 [22, 70, 71]. This relative insensitivity may be explained by the transfection of HPV-16 (human papilloma virus 16) E6/E7 oncogenes as part of the HK-2 immortalisation process, which renders these cells intrinsically resistant to cytostatic compounds.

To assess whether the anti-proliferative effect of TGF-β1 on the FPTEC cultures was associated with cell death, pro-apoptotic signalling was quantified by measuring the activities of two apoptosis effector proteases, caspase 3 and caspase 7. This revealed that 10ng/ml TGF-β1 induced a small increase in apoptotic signalling in the HK-2 cells, and a larger increase in the FPTEC. Excessive or imbalanced apoptosis within the renal tubule after injury is currently thought to be a key determinant of organ dysfunction and subsequent development of fibrosis, rather than regeneration by epithelial proliferation [72]. Previous studies have reported variable effects of TGF-β1 on apoptosis in tubular epithelial cells *in vitro*, depending on the concentration, incubation time and cell type used, with some indication that immortalised cell lines are more resistant to the effect [22, 71, 73, 74]. This difference in sensitivity was apparent in the present study, with apoptotic activity higher in the FPTEC than HK-2. This may be due to alterations in cellular phenotype intrinsic to the HK-2 immortalisation process as discussed previously.

There were several limitations to the current study that should be considered when interpreting the results. Firstly, recombinant human TGF-β1 was used in experiments on all cell types, as feline TGF-β1 is not commercially available. TGF-β1 is a well-conserved gene [75], but receptor-ligand kinetics may have been affected by this different species of origin. Secondly, whilst HREC are a more suitable comparator than the HK-2 cells, which have been immortalised, not all experiments could be carried out on all three cell types due to the limited population doublings of primary HREC. Additionally, in experiments examining Smad 2 phosphorylation status, no cross-reactive total Smad antibody could be validated for use with feline cells, and β-actin was utilised as an alternative control. The FPTEC were also obtained from cats of different ages, which were euthanized for a variety of non-renal pathologies, and so the resulting cells may have originated from very different renal microenvironments. These factors were largely unavoidable due to the need to isolate these cells from pet cats euthanized for welfare reasons. Finally, the *in vitro* cell culture environment is significantly different from the three dimensional kidney architecture, within which cells reside in complex structures. Therefore, these results obtained *in vitro* do need to be translated with caution into the whole animal.

In conclusion, we have isolated FPTEC from kidney tissue using a simple methodology, and characterised these cells alongside primary HREC and the HK-2 cell line. FPTEC retain certain characteristics of proximal tubular cells and appear similar to HREC and HK-2, indicating that these cells represent an *in vitro* model for the investigation of the effects of various insults on the feline renal tubule. In addition, TGF-β1 induced alterations suggestive of partial EMT, growth arrest and increased apoptotic signalling in FPTEC, mediated via the TGF-β1RI (ALK5)/TGF-β1RII receptor complex. Comparable results were obtained in experiments performed on HREC and HK-2, although HK-2 appeared resistant to TGF-β1-induced growth arrest. Collectively, these results suggest that TGF-β1 may be involved in the pathogenesis of renal fibrosis in cats, as in other species, and that further investigation of the cat as a naturally occurring model of pro-fibrotic mechanisms involved in human CKD may be warranted.

## Supporting information

S1 Table Primary and secondary antibodies used for immunofluorescence and western blotting. (DOCX)

S2 Table Primer sequences and optimized cycling conditions. * Primer obtained from previous publication: Penning, L. C., et al. (2007). "A validation of 10 feline reference genes for gene expression measurements in snap-frozen tissues." Vet Immunol Immunopathol 120(3–4): 212–222. ** Primer obtained from previous publication: Nguyen Van, N., et al. (2006). "Measurement of cytokine mRNA expression in intestinal biopsies of cats with inflammatory enteropathy using quantitative real-time RT-PCR." Vet Immunol Immunopathol 113(3–4): 404–414. ^$^Primer sourced from Primerdesign Ltd.
(DOCX)

S1 Fig Distribution of marker proteins in feline tissue by immunofluorescence histochemistry. Immunofluorescence staining of healthy feline tissue, counterstained with DAPI (blue) as a nuclear stain. (A, B) Distal tubules and collecting ducts were intensely positive for cytokeratin AE1/AE3 expression (green) but proximal tubules were consistently negative. (C) Glomeruli, parietal epithelial cells, scattered interstitial cells and endothelial cells were positive for vimentin expression (green). (D) Renal cortex, distal tubules demonstrated strong α-klotho expression (red), with proximal tubules demonstrating weaker expression and glomeruli negative. (E) Renal medulla interstitial capillaries demonstrated vWF expression (red). (F) Renal cortex desmin expression was not detected. (G) Bladder wall striated and smooth muscle demonstrated intense desmin expression. (H, I) Mouse isotype and rabbit isotype controls respectively were both negative. Images are representative of results obtained from tissue derived from three cats.
(TIF)

S2 Fig Immunoblotting for detection of marker proteins in cell lysates. Immunoblots of FPTEC lysates from three separate isolations. (A) FPTEC show consistent expression of the epithelial marker cytokeratin AE1/AE3 and tubular marker α-Klotho, alongside the mesenchymal marker vimentin. HK-2 cell lysate was used as a positive control. (B) FPTEC do not express the endothelial cell marker vWF. Human umbilical vein endothelial cell lysate (HUVEC) was used as a positive control.
(TIF)
