# Supplementary material for: The cat as a naturally occurring model of renal interstitial fibrosis: Characterisation of primary feline proximal tubular epithelial cells and comparative pro-fibrotic effects of TGF-β1
Source: PLoS One. 2018 Aug 23;13(8):e0202577. doi: 10.1371/journal.pone.0202577 (PMC6107233; doi:10.1371/journal.pone.0202577)
Supplement: S1 Table — (DOCX) [file pone.0202577.s001.docx]

**S1** Table: Primary antibodies used for immunofluorescence and western blotting

| Antibody | Supplier | Secondary antibody | Dilution for IHC/ICC | Dilution for western blot | Incubation temperature for western blot |
| --- | --- | --- | --- | --- | --- |
| Cytokeratin AE1/AE3 | Dako | Mouse | 1:100 | 1:1000 | 4°C |
| Vimentin | Dako | Mouse | 1:100 | 1:1000 | 4°C |
| Desmin | Dako | Mouse | 1:100 | N/A | N/A |
| α-klotho | Abcam | Rabbit | 1:50 | 1:500 | 4°C |
| CD29 | Bio-rad | Mouse | 1:100 | 1:500 | 4°C |
| CD44 | ABD serotec | Rat | 1:100 | N/A | N/A |
| vWF | Dako | Rabbit | 1:200 | 1:1000 | 4°C |
| VCAM-1 | R&D | Mouse | 1:100 | N/A | N/A |
| Rabbit Polyclonal Isotype control | Biolegend | Rabbit | 1:50 – 1:200 | N/A | N/A |
| Mouse IgG1 κ isotype control | Biolegend | Mouse | 1:50 – 1:200 | N/A | N/A |
| β-actin | Sigma-Aldrich | Mouse | N/A | 1:5000 | 4°C |
| pERK (T202/T204) | Cell signalling | Rabbit | N/A | 1:1000 | Room temp |
| ERK 1 | Santa Cruz | Rabbit | N/A | 1:5000 | Room temp |
| Caspase 3 | Cell signalling | Rabbit | N/A | 1:1000 | 4°C |
| Cleaved caspase 3 | Cell signalling | Rabbit | N/A | 1:1000 | 4°C |
| pSMAD 2 | Cell signalling | Rabbit | N/A | 1:1000 | 4°C |

Secondary antibodies used for immunofluorescence and western blotting

| Antibody | Supplier | Application | Conjugate |
| --- | --- | --- | --- |
| Goat Anti-Rabbit IgG | Pierce | Western blot | HRP |
| Goat Anti-Mouse IgG | Pierce | Western blot | HRP |
| Goat Anti-Mouse IgG H&L | Abcam | Immunofluorescence | Alexa Fluor 488 |
| Goat Anti-Rabbit IgG H&L | Abcam | Immunofluorescence | Alexa Fluor 594 |
