# Supplementary material for: The cat as a naturally occurring model of renal interstitial fibrosis: Characterisation of primary feline proximal tubular epithelial cells and comparative pro-fibrotic effects of TGF-β1
Source: PLoS One. 2018 Aug 23;13(8):e0202577. doi: 10.1371/journal.pone.0202577 (PMC6107233; doi:10.1371/journal.pone.0202577)
Supplement: S2 Table — * Primer obtained from previous publication: Penning, L. C., et al. (2007). "A validation of 10 feline reference genes for gene expression measurements in snap-frozen tissues." Vet Immunol Immunopathol 120(3–4): 212–222. ** Primer obtained from previous publication: Nguyen Van, N., et al. (2006). "Measurement of cytokine mRNA expression in intestinal biopsies of cats with inflammatory enteropathy using quantitative real-time RT-PCR." Vet Immunol Immunopathol 113(3–4): 404–414. $Primer sourced from Primerdesign Ltd. (DOCX) [file pone.0202577.s002.docx]

S2 Table: Primer sequences and optimized cycling conditions

**Feline Primers**

| **Gene** | **Primer sequence (forward primer first, 5’ to 3’)** | **Amplicon size (bp)** | **GC**  **Content (%)** | **Annealing temp** | **Primer dilution** |
| --- | --- | --- | --- | --- | --- |
| *RPS7** | GTCCCAGAAGCCGCACTTTGAC  CTCTTGCCCACAATCTCGCTCG | 82 | 59  59 | 60.8 | **1:10** |
| *GAPDH** | AGTATGATTCCACCCACGGCA  GATCTCGCTCCTGGAAGATGGT | 101 | 52 55 | 64.1 | **1:10** |
| *COL1A1* | GAGAGCATGACCGACGGATT  TAGGTGATGTTCTGGGACGC | 122 | 55  55 | 58.7 | **1:10** |
| *TGFB1*** | GGAATGGCTGTCCTTTGATG  TGCAGTGTGTTATCTTTGCTGTC | 120 | 50  43.5 | 60 | 1:10 |
| *CTGF* | GGAAGACACATTTGGCCCAG  GCTTCTCCAACCTGCAGAAG | 146 | 55  55 | 62.1 | **1:10** |
| *TG-2* | TACAAGTACCCGGAGGGGTC  CGAAGACGTCGAAGTCGCTA | 151 | 60  55 | 56.4 | 1:10 |
| *CDH1* | TACATTCTGTACTCTCATGCTGT  AGCGCTTTCCATGACAGAAC | 144 | 39.1  50 | 59.4 | **1:10** |
| *CDH2* | TCCTTGCTTCTGACAATGGAATC  TCAAAAGCAAATGGTCCAGCA | 196 | 43.5  42.9 | 60.1 | **1:10** |
| *FN1* | CCCTCACCAATCTCACTCCA  CCCTCGGAACATCAGAAACTG | 117 | 55  52.4 | 58 | **1:10** |
| *S100A4* | TTAGGGAAAAGGACGGATGATGC  CGACACAGGACAGGAAGACA | 112 | 47.8  55 | 60.1 | **1:10** |

**Human Primers**

| **Gene** | **Primer sequence (forward primer first, 5’ to 3’)** | **Amplicon size (bp)** | **GC**  **Content (%)** | **Annealing temp** | **Primer dilution** |
| --- | --- | --- | --- | --- | --- |
| *RPS7* | TCGTCTTTATCGCTCAGAGGA  GGCTGCCATCTAGTTTGACG | 107 | 47.6  55 | 61 | 1:10 |
| *GAPDH* | TCCTCTGACTTCAACAGCGACAC  CACCCTGTTGCTGTAGCCAAATTC | 126 | 52 55 | 63.3 | 1:20 |
| *COL1A1*^$^ | AGACAGTGATTGAATACAAAACCA  GGAGTTTACAGGAAGCAGACA | 130 | 33.3  47.6 | 55.8 | 1:20 |
| *TGFB1* | TACAGCAACAATTCCTGGCG  AAGCCCTCAATTTCCCCTCC | 196 | 50 55 | 60.7 | 1:20 |
| *CTGF* | GGAAGAGAACATTAAGAAGGGCA  CCGTCGGTACATACTCCACA | 132 | 43.5  55 | 59.4 | 1:10 |
| *TG-2* | GAGCGAGATGATCTGGAACTTC  GCATCGTACTTGGTGCTCAG | 189 | 50 55 | 60.7 | 1:10 |
| *CDH1*^$^ | CATGAGTGTCCCCCGGTATC  CAGTATCAGCCGCTTTCAGA | 89 | 60 50 | 59 | 1:10 |
| *CDH2* | AAACCTTTCACTGCGGTACA  TGTGCCCTCAAATGAAACCG | 147 | 45  50 | 57 | 1:10 |
