# Supplementary figures and images for: The cat as a naturally occurring model of renal interstitial fibrosis: Characterisation of primary feline proximal tubular epithelial cells and comparative pro-fibrotic effects of TGF-β1
Source: PLoS One. 2018 Aug 23;13(8):e0202577. doi: 10.1371/journal.pone.0202577 (PMC6107233; doi:10.1371/journal.pone.0202577)

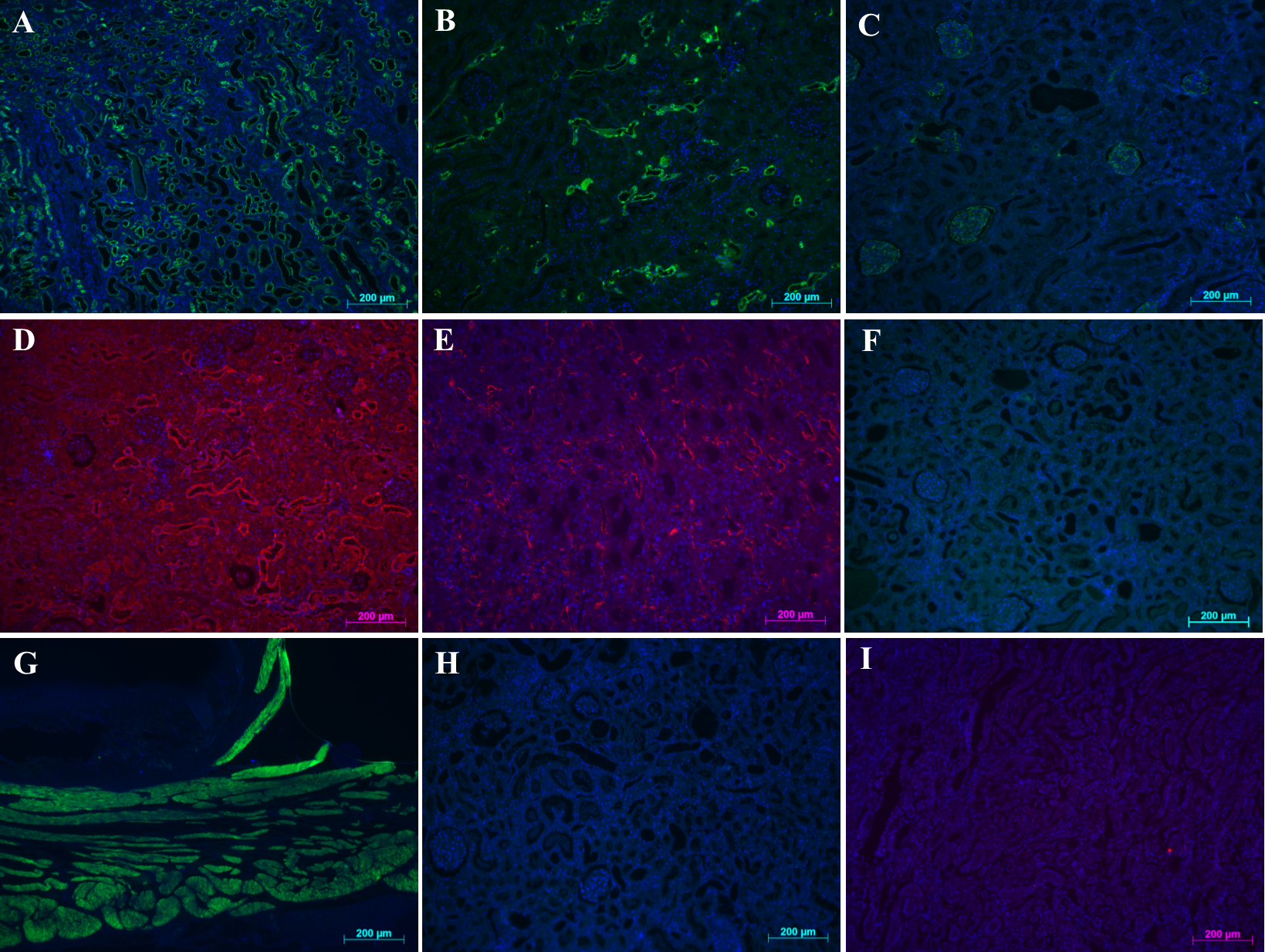

Supplement: S1 Fig — Immunofluorescence staining of healthy feline tissue, counterstained with DAPI (blue) as a nuclear stain. (A, B) Distal tubules and collecting ducts were intensely positive for cytokeratin AE1/AE3 expression (green) but proximal tubules were consistently negative. (C) Glomeruli, parietal epithelial cells, scattered interstitial cells and endothelial cells were positive for vimentin expression (green). (D) Renal cortex, distal tubules demonstrated strong α-klotho expression (red), with proximal tubules demonstrating weaker expression and glomeruli negative. (E) Renal medulla interstitial capillaries demonstrated vWF expression (red). (F) Renal cortex desmin expression was not detected. (G) Bladder wall striated and smooth muscle demonstrated intense desmin expression. (H, I) Mouse isotype and rabbit isotype controls respectively were both negative. Images are representative of results obtained from tissue derived from three cats. (TIF) [file pone.0202577.s003.tif]

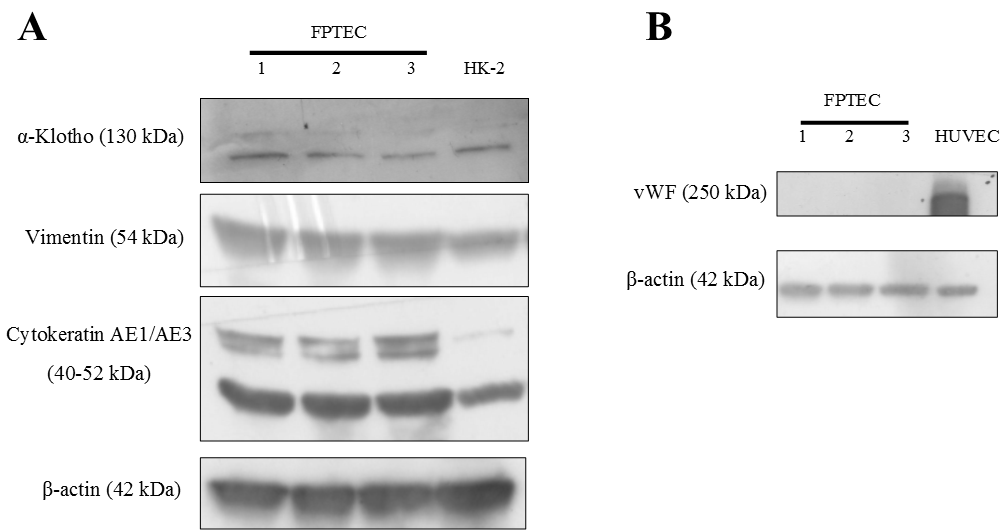

Supplement: S2 Fig — Immunoblots of FPTEC lysates from three separate isolations. (A) FPTEC show consistent expression of the epithelial marker cytokeratin AE1/AE3 and tubular marker α-Klotho, alongside the mesenchymal marker vimentin. HK-2 cell lysate was used as a positive control. (B) FPTEC do not express the endothelial cell marker vWF. Human umbilical vein endothelial cell lysate (HUVEC) was used as a positive control. (TIF) [file pone.0202577.s004.tif]
